# The association of leptin and adiponectin with hepatocellular carcinoma risk and prognosis: a combination of traditional, survival, and dose-response meta-analysis

**DOI:** 10.1186/s12885-020-07651-1

**Published:** 2020-11-30

**Authors:** Lilong Zhang, Qihang Yuan, Man Li, Dongqi Chai, Wenhong Deng, Weixing Wang

**Affiliations:** 1 Department of General Surgery, Renmin Hospital of Wuhan University, Wuhan, 430060 Hubei China; 2 Department of General Surgery, The First Affiliated Hospital of Dalian Medical University, Dalian, 116011 Liaoning China

**Keywords:** Leptin, Adiponectin, Hepatocellular carcinoma, Meta-analysis

## Abstract

**Background:**

An increasing number of studies have focused on the association between leptin, adiponectin levels and the risk as well as the prognosis of hepatocellular carcinoma. However, the reported results are conflicting.

**Methods:**

A meta-analysis was performed to assess the correlation between leptin, adiponectin levels and risk and prognosis of hepatocellular carcinoma (CRD42020195882). Through June 14, 2020, PubMed, Cochrane Library and EMBASE databases were searched, including references of qualifying articles. Titles, abstracts, and main texts were reviewed by at least 2 independent readers. Stata 16.0 was used to calculate statistical data.

**Results:**

Thirty studies were included in this meta-analysis and results showed that hepatocellular carcinoma group had significantly higher leptin levels than the cancer-free control group (SMD = 1.83, 95% CI (1.09, 2.58), *P* = 0.000), the healthy control group (SMD = 4.32, 95% CI (2.41, 6.24), *P* = 0.000) and the cirrhosis group (SMD = 1.85, 95% CI (0.70, 3.01), *P* = 0.002). Hepatocellular carcinoma group had significantly higher adiponectin levels than the healthy control group (SMD = 1.57, 95% CI (0.37, 2.76), *P* = 0.010), but no statistical difference compared with the cancer-free control group (SMD = 0.24, 95% CI (− 0.35, 0.82), *P* = 0.430) and the cirrhosis group (SMD = − 0.51, 95% CI (− 1.30, 0.29), *P* = 0.213). The leptin rs7799039 polymorphism was associated with increased risk of hepatocellular carcinoma (G vs A: OR = 1.28, 95% CI (1.10, 1.48), *P* = 0.002). There were linear relationships between adiponectin levels and the risk of hepatocellular carcinoma (OR = 1.066, 95% CI (1.03, 1.11), *P* = 0.001). In addition, the results showed that high/positive expression of adiponectin was significantly related to lower overall survival in hepatocellular carcinoma patients (HR = 1.70, 95% CI (1.22, 2.37), *P* = 0.002); however, there was no significantly association between the leptin levels and overall survival (HR = 0.92, 95% CI (0.53, 1.59), *P* = 0.766).

**Conclusion:**

The study shows that high leptin levels were associated with a higher risk of hepatocellular carcinoma. Adiponectin levels were proportional to hepatocellular carcinoma risk, and were related to the poor prognosis.

## Introduction

Hepatocellular carcinoma (HCC) accounts for the sixth most common cancer in the world and the fourth leading cause of cancer death globally [1], and has highly invasive and aggressive behavior [2]. Generally, HCC is secondary to liver cirrhosis or viral hepatitis. About 80% of patients newly infected with the virus will develop chronic infections, and 10–20% of them will develop liver cirrhosis within 20–30 years, and 1–5% of them will meet advanced liver cancer [3]. Studies have further identified obesity, particularly abdominal obesity, as a potential risk factor for HCC. Therefore, leptin and adiponectin, as the most plenteous and the best-studied obesity-related adipokines, may play an important role in the development of HCC [4, 5].

Leptin is well known as a regulator, which acts in energy expenditure and food intake. Research on leptin has shown that it not only plays a key role in metabolism, but also mediates tumor development by enhancing tumor angiogenesis, promoting cell proliferation, migration, invasion, and inhibiting tumor cell apoptosis [6]. Leptin acts on receptors expressed in many tissues, including the liver. Adiponectin, also known as AdipoQ, was first described by Arita et al. It has liver-protecting and anti-inflammatory properties [7, 8]. The beneficial effects of AdipoQ have been observed in alcoholic and non-alcoholic liver disease models [9]. Animal studies also proven that AdipoQ has a protective effect in liver cancer and cirrhosis [10, 11], and high AdipoQ levels in patients with cirrhosis and HCC are related to disease progression [12, 13].

In recent years, reports on the correlation between leptin, AdipoQ levels and HCC have gradually increased, and some studies have tried to clarify the role of the two in HCC. Unfortunately, the reported results are conflicting. Therefore, we conducted a meta-analysis to systematically determine the relationship between leptin, AdipoQ levels and HCC risk and prognosis.

## Methods

The protocol for this meta-analysis is available in PROSPERO (CRD42020195882).

### Literature search strategy

The PubMed, EMBASE, and Cochrane Library Databases were explored on June 14, 2020, limited to the English language. The following terms were searched in [Title/Abstract]: Adiponectin [MeSH], AdipoQ, Adipocyte Complement Related Protein 30 kDa, Adipose Most Abundant Gene Transcript 1, apM 1 Protein, ACRP30 Protein, Adipocyte, C1q and Collagen Domain Containing Protein, Leptin [MeSH], Obese Protein, Obese Gene Product, Ob Gene Product, Ob Protein, Liver Neoplasms [MeSH], Liver Neoplasm*, Hepatocellular Cancer*, Hepatic Cancer*, Liver Cancer*, Cancer of Liver. Besides, the reference lists of included articles were manually searched.

### Study selection criteria

Inclusion criteria: (1) studies were focused on the correlation between leptin or AdipoQ levels and HCC risk, and provided full text and complete data in HCC patients and cancer-free controls (CFC), or (2) investigated the correlation between AdipoQ or leptin levels and the prognosis of HCC, and provided sufficient information to get the hazard ratio (HR) and 95% confidence intervals (CIs). Exclusion criteria: conference abstracts, case reports, comments, review, and experimental animal studies were excluded. When studies reported on the same or overlapping patient populations, only the study with the most complete data set and the most rigorous methodology was used.

### Data extraction

Basic information from each study were extracted: the first author, year of publication, country, study design, study period, number of subjects, and sample source. Also, the following information was extracted in Table 1: the source of the case group, the source of the CFC group, age, gender, body mass index, measurement indicators, and detection methods. Table 2 summarized the following information: source of cases/controls, matching variables, single nucleotide polymorphisms, genotyping methods, frequency of cases, and control genotypes. In Table 3, the following information was selected: follow-up, measured indicators, detected methods, cut-off values, survival analysis, HR sources, and analysis methods.

**Table 1:** Main characteristics of the studies examining the relationship between circulating leptin, adiponectin levels and HCC

Author, Year, Country	Study design	Study period	the source of case group	the source of Cancer-free control group	Number	Mean age	Males	BMI	Sample source	Measured indicators	Detected method	NOS score
Abouzied, 2017, Egypt	C	–	HCC	Healthy controls	25/25	57.7/29.2	18/23	21.7/22.2	Serum	Leptin	ELISA	5
Aleksandrova, 2014, Europe	N	2000–2006	HCC	Healthy controls	125/250	60.1/60.1	85/171	28.1/26.9	Serum	Leptin and AdipoQ	ELISA	8
Ataseven, 2006, Yurkey	C	–	HBV-related HCC	HBV-related cirrhosis/ Healthy controls	22/23/25	59.8/45.5/37.1	15/11/11	–	Serum	Leptin	ELISA	8
Bakir, 2017, Egypt	S	03/2016–11/2016	HCV-related cirrhotic HCC	HCV-related cirrhosis	50/40	53.2/50.7	29/25	24.5/26.1	Serum	Leptin	ELISA	5
Bastard, 2018, France	N	03/2006–07/2016	Viral cirrhotic HCC	Viral cirrhosis	56/96	59.8/58.9	34/61	25.6/25.8	Serum	Leptin and AdipoQ	ELISA	7
Chen, CL, 2014, Taiwan	N	01/1999–12/2004	HBV-related HCC	Chronic hepatitis B	187/374	52.4/52.2	154/311	–	Plasma	Leptin and AdipoQ	ELISA	8
Chen, MJ, 2012, Taiwan	C	01/2009–12/2009	Viral HCC	Healthy controls	65/165	58.8/47.7	47/112	24.7/24.4	Serum	AdipoQ	RIA	6
Costantini,2013, Italy	C	–	HCV-related HCC	HCV-related cirrhosis / Chronic hepatitis C / Healthy controls	26/30/30/20	70.0/68/63.4/60.9	18/14/15/9	–	Serum	Leptin	ELISA	8
Feder, 2019, Germany	P	05/2012–05/2015	HCC	Healthy controls	32/49	–	–	–	Serum	AdipoQ	ELISA	7
Fukushima, 2010, Japan	P	1993–2003	HCV-related HCC	Chronic hepatitis C	9/27	53.0/51.3	5/11	–	Serum	Leptin (RIA) and HMW AdipoQ (ELISA)		8
Hamdy, 2015, Egypt	S	01/2014–12/2014	HCV-related cirrhotic HCC	HCV-related cirrhosis	61/29	52.3/52.3	51/21	33.7/36.7	Serum	AdipoQ	ELISA	8
Khattab, 2012, Egypt	C	02/2009–01/2010	HCC	Chronic hepatitis C / Healthy controls	147/147/320	43.9/41.6/42.9	114/115/201	24.9/25.1/25.3	Serum	AdipoQ	ECLI	6
Kotani, 2009, Japan	N	1990–1999	HCC	Healthy controls	59/334	63.5/62.7	–	–	Serum	AdipoQ	ELISA	5
Liu, CJ, 2009, Taiwan	S	01/2002–10/2005	HBV-related HCC	HBV-related cirrhosis / Chronic hepatitis B/ Healthy controls	120/40/120/116	50.7/50.3/30/53.8	100/29/63/67	–	Serum	AdipoQ	ELISA	8
Liu, ZW, 2005, China	C	–	HCV-related cirrhotic HCC	HCV-related cirrhosis/ Chronic hepatitis C/ Healthy controls	2/10/30/30	59.5/53.7/41/39.4	2/6/17/18	23.0/22.7/23.0/23.1	Serum	Leptin	ELISA	8
Michikawa, 2013, Japan	N	1993–2006	Viral HCC	Chronic viral hepatitis	90/117	–	62/80	–	plasma	AdipoQ	ELISA	8
Radwan, 2019, Egypt	S	–	HCC	Chronic hepatitis C	48/52	53.2/52.5	26/32	25.2/27.7	Serum	AdipoQ	ELISA	5
Sadik, 2012, Egypt	C	01/2008–02/2009	HCV-related HCC	HCV-related cirrhosis/ Healthy controls	69/36/21	59.1/53.0/55.8	43/23/13	28.0//27.1/29.1	Serum	Leptin and AdipoQ	ELISA	6
Sumie, 2011, Japan	C	01/1997–10/2007	HCV-related HCC	Chronic hepatitis C	97/97	67.4/61.2	67/67	22.5/23.1	Serum	AdipoQ	ELISA	7
Voumvouraki, 2011, Greece	C	–	Viral cirrhotic HCC	Viral cirrhosis/ Chronic hepatitis C / Healthy controls	38/34/44/60	62.0/60.0/53.0/−−	25/12/8/−−	–	Serum	Leptin	ELISA	8
Wang, 2003, Taiwan	C	01/2000–12/2000	Viral cirrhotic HCC	Viral cirrhosis/ Healthy controls	31/26/25	65.0/59.0/65.0	31/26/25	23.2/23.7/24.4	Serum	Leptin	RIA	6

*P* Cohort, *S* Cross-sectional, *C* Case-control, *N* Nested Case-control, *HCC* Hepatocellular carcinoma, *HCV* hepatitis C virus, *HBV* hepatitis B virus, *ELISA* Enzyme-linked immunosorbent assay, *ECLI* Electro-chemiluminescence immunoassay, *RIA* Radioimmunoassay, *HMW AdipoQ* High Molecular Weight Adiponectin, *BMI* body mass index, *NOS* Newcastle-Ottawa Scale

**Table 2:** Main characteristics of the studies examining the relationship between leptin, adiponectin gene polymorphism and HCC

Author, Year, Country	Study design	Case/control	Number	Matching variables	SNP	Samples source	Genotyping methods	Frequency of case genotype	Frequency of control genotype	NOS score
Amer, 2017, Egypt	C	HCC/Healthy controls	150/100	age, sex and smoking rate	rs7799039 (leptin gene)	blood	PCR-RFLP technique	AA = 60; AG = 69; GG = 21; G allele =111; A allele =189	AA = 49; AG = 47; GG = 4; G allele =54; A allele =146	7
Zhang, 2018, China	C	HCC/Healthy controls	575/921	age and sex	rs7799039 (leptin gene)	blood	SNPscan™	AA = 295; AG = 221; GG = 59; G allele = 339; A allele =811	AA = 505; AG = 360; GG = 56; G allele = 472; A allele =1370	7
Cai, 2013 China	C	HCC/Healthy controls	200/200	age and sex	rs1501299 (adiponectin gene)	blood	DNA sequencing	TT = 12; TG = 60; GG = 128; G allele = 316; T allele =84	TT = 39; TG = 69; GG = 92; G allele = 253; T allele =147	8

*C* Case-control, *SNP* single-nucleotide polymorphisms, *PCR-RFLP* polymerase chain reaction-restriction fragment length polymorphism, *NOS* Newcastle-Ottawa Scale

**Table 3:** Main characteristics of the studies examining the relationship between adiponectin, leptin levels and the prognosis of HCC

Author, Year, Country	Study design	Study period	Number	Follow-up (months)	Sample	Measured indicators	Detected method	Cut-off value	Survival analysis	Source of HR	Analytic method	NOS score
Siegel, 2014, USA	P	2008–2012	140	8	Serum	AdipoQ; Leptin	RIA	≥13.05 μg/ml vs<13.05 μg/ml; ≥7.9 ng/ml vs<7.9 ng/ml	OS	Report	M;U	7
Shen, 2016, USA	P	2008–2014	135	84	Plasma	AdipoQ	ELISA	≥13.10 μg/ml vs<13.10 μg/ml	OS	Report	M	7
Shin, 2014, Korea	P	1996–2001	75	82.5	Cytoplasmic	AdipoQ	IHC	Positive: > 0% of cells stained vs Negative: no cells stained	OS	Report	M	6
Watanabe, 2011, Japan	P	2006–2008	33	39	Plasma	Leptin	NA	≥5.0 ng/ml vs<5.0 ng/ml	DFS	Report	M	7
Wang, 2014, Taiwan	P	1999–2003	85	NA	Cytoplasmic	AdipoQ	IHC	Low: - ~ + vs High: ++ ~ +++^a^	OS	SC	M	7
Wang, 2006, Taiwan	P	1994–2003	68	31.7	Cytoplasmic	Leptin	IHC	Low: - ~ + vs High: ++ ~ +++^a^	OS	SC	M	6

*ELISA* Enzyme-linked immunosorbent assay, *RIA* Radioimmunoassay, *IHC* Immunohistochemistry, *NA* Not available, *DFS* Disease-free survival, *OS* Overall survival, *SC* Survival curves, *U* Univariate, *M* Multivariate. ^a^Semiquantitative scoring system, *NOS* Newcastle-Ottawa Scale

### Quality assessment

The Newcastle-Ottawa Scale was used to assess the quality of non-randomized controlled studies. The following three factors were considered: patient selection, comparability of study groups, and outcome evaluation. The highest score was 9 points, and studies with a score ≥ 7 were considered high-quality [14]. The above steps were independently cross-checked by two researchers (Zhang Lilong, Yuan Qixing), and all differences were handled by the senior author (Wang Weixing).

### Statistical analysis

Stata 16.0 was used for statistical analysis. Dichotomous variables and continuous variables were compared by the odds ratio (OR) and standardized mean difference (SMD). The hazard ratio (HR) was calculated to assess the correlation between AdipoQ or leptin expression and the prognosis of HCC. In the case that the study only provided the Kaplan-Meier survival curve, used Engage Digitizer version 2.11 software to extract relevant values from the survival curve and calculate HR (95% CI) [15, 16]. For all analyses, the 95% confidence interval (CI) was used. *P*<0.05 was defined as statistically significant. The chi-squared test was calculated to evaluate the statistical heterogeneity between different studies. *P* > 0.1 and I^2^ < 50% indicated low heterogeneity where a fixed-effect model was used; otherwise, the random-effect model was adopted.

Furthermore, a 2-stage dose-response meta-analysis was performed to explore the association between different categories of leptin, AdipoQ levels and HCC risk [17, 18]. 1) The fixed-effect nonlinear model was constructed based on the restrictive cubic spline function (Knot =3). 2) According to the results of the heterogeneity test and nonlinear correlation test, the corresponding model was adopted.

Finally, for indicators with high heterogeneity, we conducted a sensitivity analysis by leaving one out method to check the robustness of the results and determine the source of heterogeneity. Besides, meta-regression analysis was performed to explore the potential sources of heterogeneity. For indicators containing more than 10 articles, we generated a funnel plot to visually check publication bias. Begg’s and Egger’s tests were also performed to quantitatively analyze publication bias, and *P < 0.05* was considered statistically significant. If necessary, we verify the results of the publication bias by establishing a trim and fill funnel plot.

## Results

### Studies retrieved and characteristics

**Fig. 1:**
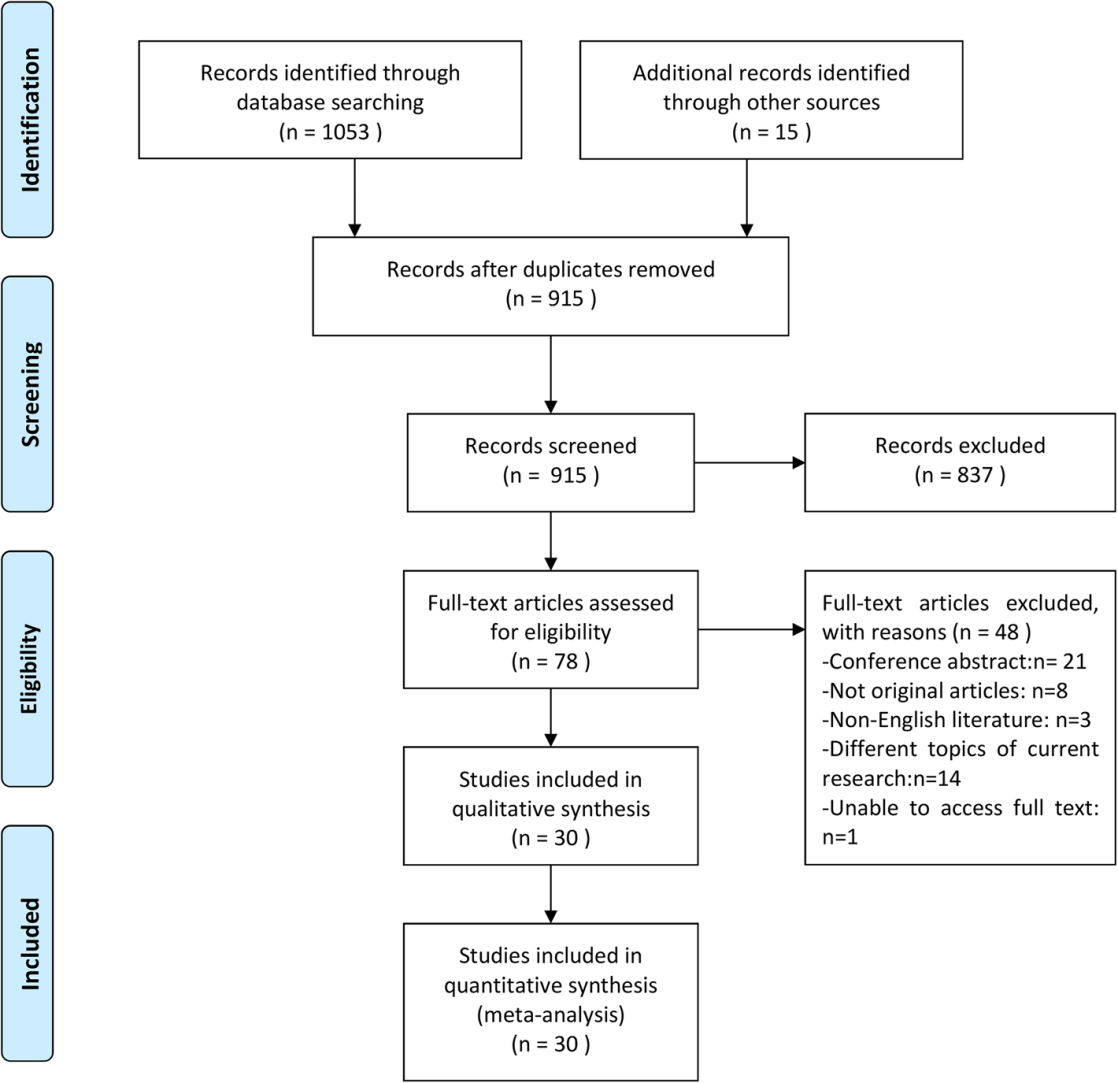
Flow diagram of the meta-analysis search process

In this meta-analysis, we identified 1068 potentially eligible records and screened their titles and abstracts for inclusion. After reading the full text of 78 records in detail, 30 studies met our inclusion criteria (Fig. 1). Although Ebrahim’s article fits the research topic, it is excluded because the full text is not available [19]. Twenty-one articles [20–40] (10 case-control, 5 nested case-control, 4 cross-sectional, and 2 cohort studies) evaluated the relationship between Leptin or AdipoQ levels and HCC risk, and main characteristics were summarized in Table 1. Three case-control studies [41–43] assessed the relationship between Leptin or AdipoQ gene polymorphism and HCC risk, and main characteristics were reported in Table 2. Six articles [44–49] analyzed the relationship between AdipoQ or leptin expression and the prognosis of HCC, and the main characteristics were summarized in Table 3. Quality assessment of the included studies using the Newcastle–Ottawa scale was shown in Tables 1, 2, 3, and the scores ranged from 5 to 8. Twenty articles were awarded 7 or 8 points, and considered as high-quality; Six studies were awarded 6 points and four studies were awarded 5 points, which were considered as moderate quality.

### Association between circulating leptin levels and HCC risk

**Fig. 2:**
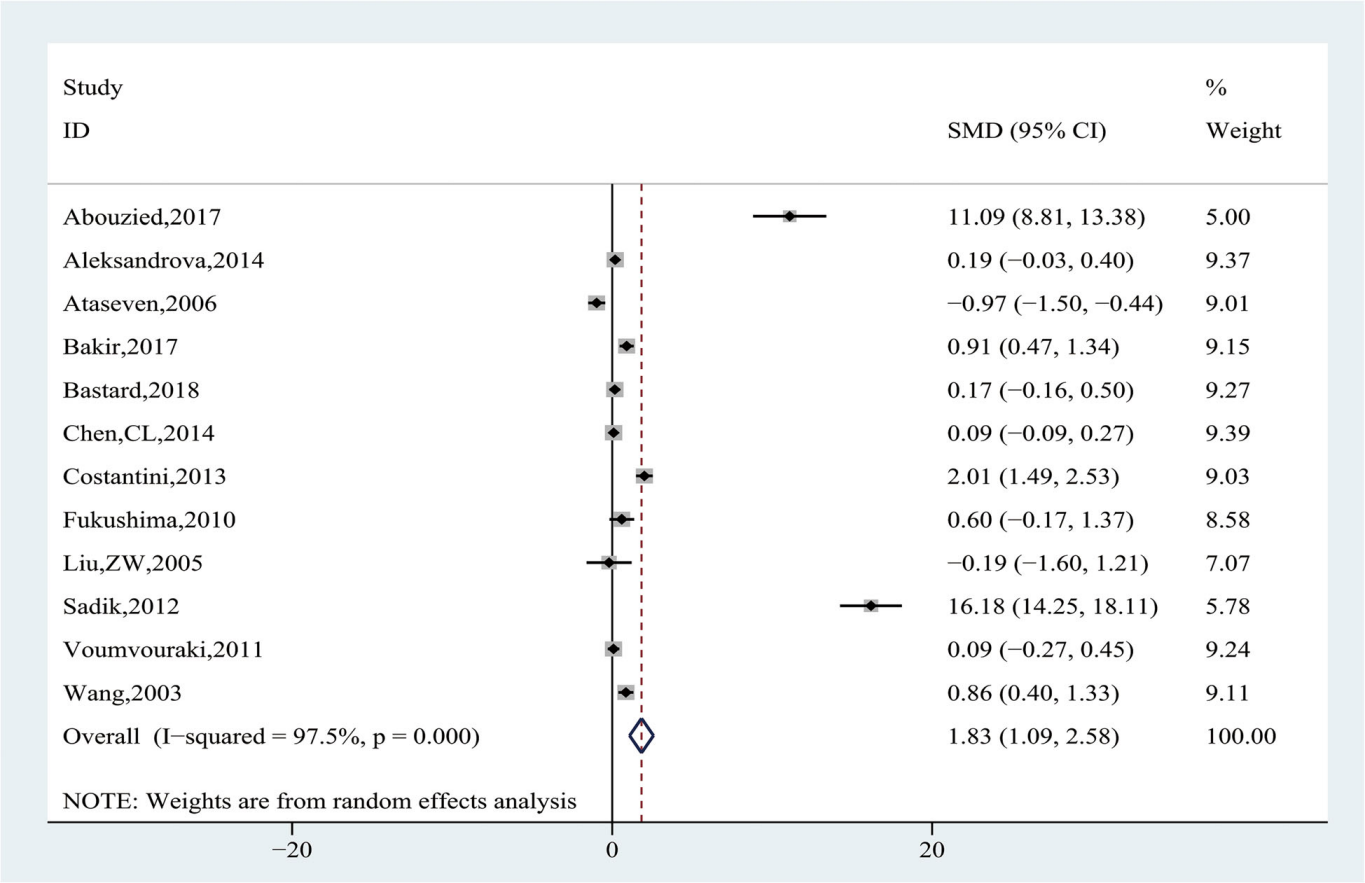
Forest plot of comparing circulating leptin levels between the HCC and cancer-free control group

**Fig. 3:**
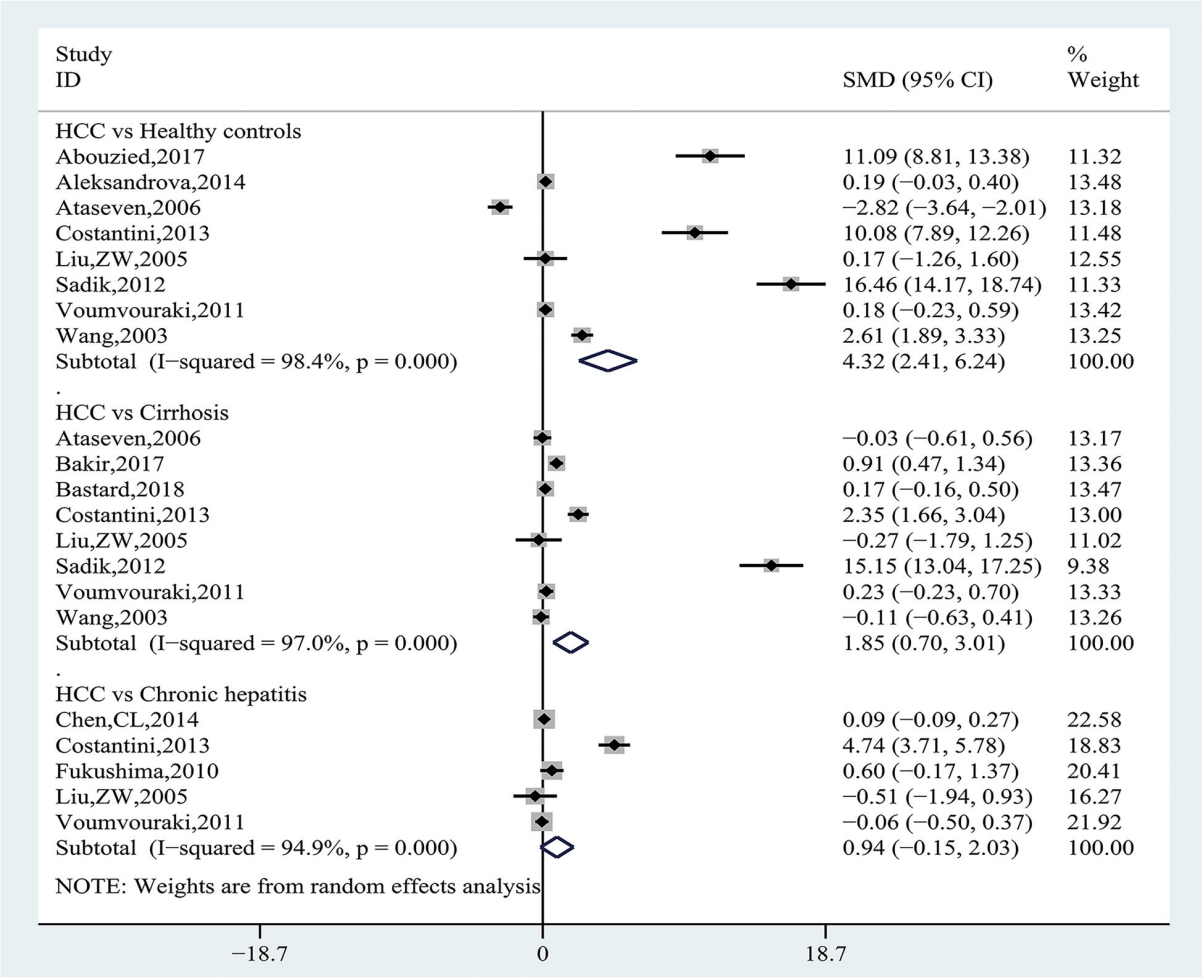
Forest plot of the subgroup analyses concerning circulating leptin levels

Pooling data of 12 studies [20–25, 27, 29, 34, 37–39] with 1896 participants assessed the association between leptin levels and HCC risk. Heterogeneity analysis showed that significant heterogeneity was observed among the studies (I^2^ = 97.5%, *P* = 0.000), and the random-effect model was applied. The results showed that leptin levels were significantly higher in the HCC group than CFC group (SMD = 1.83, 95% CI (1.09, 2.58), *P* = 0.000) (Fig. 2). Subgroup analysis, according to the source of CFC group, showed HCC group had significantly higher leptin levels than the healthy control group (SMD = 4.32, 95% CI (2.41, 6.24), *P* = 0.000) and the cirrhosis group (SMD = 1.85, 95% CI (0.70, 3.01), *P* = 0.002), but there was no statistical difference when compared with the chronic hepatitis group (SMD = 0.94, 95% CI (− 0.1, 2.03), *P* = 0.090) (Fig. 3 and Table 4). We further conducted subgroup analysis by the source of case group, and the results showed HCV-related cirrhotic HCC had significantly higher leptin levels than HCV-related cirrhosis (SMD = 0.82, 95% CI (0.40, 1.24), *P* = 0. 0.000), whereas there was no difference in other subgroups (Table 4).

**Table 4:** Subgroup analysis of the association between circulating leptin levels and HCC risk

Variable	Included studies	Test of association			Test of heterogeneity		
		SMD	95%CI	*P*-value*	Modal	*P*-value	I^2^
HCC vs Healthy controls							
Overall	[23–25, 30, 37, 40–42]	4.32	2.41, 6.24	**0.000**	RE	0.000	98.4%
HCC(unreported reason) vs Healthy controls	[23, 24]	5.58	−5.11, 16.26	0.306	RE	0.000	98.8%
Viral cirrhotic HCC vs Healthy controls	[37, 41, 42]	1.02	− 0.78, 2.79	0.263	RE	0.000	94.1%
HCV-related HCC vs Healthy controls	[30, 37, 40]	8.87	− 1.08, 1.82	0.081	RE	0.000	98.7%
HCC vs Cirrhosis							
Overall	[25–27, 30, 37, 40–42]	1.85	0.70, 3.01	**0.002**	RE	0.000	97.0%
HCV-related cirrhotic HCC vs HCV-related cirrhosis	[26, 37]	0.82	0.40, 1.24	**0.000**	FE	0.145	53.0%
HCV-related HCC vs HCV-related cirrhosis	[30, 40]	8.71	−3.84, 21.25	0.174	RE	0.000	99.2%
Viral cirrhotic HCC vs Viral cirrhosis	[27, 41, 42]	0.13	−0.11,0.37	0.302	FE	0.591	0
HCC vs Chronic hepatitis							
Overall	[28, 30, 32, 37, 41]	0.94	−0.15, 2.03	0.090	RE	0.000	94.9%
HCV-related HCC vs Chronic hepatitis C	[30, 32, 37]	1.63	−1.39, 4.65	0.290	RE	0.000	96.0%
Viral cirrhotic HCC vs Chronic hepatitis C	[37, 41]	−0.10	− 0.51,0.32	0.643	FE	0.561	0
Ethnicity							
Asian	[25, 28, 32, 37, 42]	0.10	−0.50, 0.70	0.751	RE	0.000	85.6%
Caucasian	[24, 27, 30, 41]	0.58	−0.06, 1.22	0.077	RE	0.000	93.3%
African	[23, 26, 40]	9.36	−1.27, 19.99	0.084	RE	0.000	99.3%
Sample size							
< 100	[23, 25, 26, 32, 37, 42]	1.57	0.22, 2.91	**0.022**	RE	0.000	95.8%
≥ 100	[24, 27, 28, 30, 40, 41]	2.23	1.21, 3.26	**0.000**	RE	0.000	98.4%
Mean age							
< 60	[23, 25–28, 32, 37, 40]	2.87	1.57, 4.17	**0.000**	RE	0.000	98.2%
≥ 60	[24, 30, 41, 42]	0.76	0.03, 1.49	**0.040**	RE	0.000	93.7%
Study design							
Case-control	[23, 25, 30, 37, 40, 41]	3.81	1.83, 5.79	**0.000**	RE	0.000	97.5%
Nested Case-control	[24, 27, 28]	0.14	0.01, 0.26	**0.035**	FE	0.777	0.0%
Assay method							
ELISA	[23–28, 30, 37, 40, 41]	2.13	1.27, 2.99	**0.000**	RE	0.000	97.9%
RIA	[32, 42]	0.79	0.39, 1.19	**0.000**	FE	0.570	0.0%
Alanine aminotransferase							
< 70 U/L	[23, 26, 28, 32, 37, 40]	4.42	2.26, 6.50	**0.000**	RE	0.000	98.6%
≥ 70 U/L	[25, 27, 30, 41, 42]	0.43	−0.38, 1.23	0.296	RE	0.000	94.4%
Albumin							
< 3.5 g/dl	[25, 26, 30, 40, 42]	3.47	1.28, 5.66	**0.002**	RE	0.000	98.7%
≥ 3.5 g/dl	[27, 28, 32, 41]	0.12	−0.02, 0.26	0.091	FE	0.633	0.0%

*RE* Random-effects model, *FE* Fixed-effects model, *HCC* Hepatocellular carcinoma, *HCV* hepatitis C virus

*Statistically significant results were shown in bold

In addition, we also performed other subgroup analyses and the results were shown in Table 4. Stratification by ethnicity showed no significant difference in the HCC group and CFC group in Asian (SMD = 0.10, 95% CI (− 0.50, 0.70), *P* = 0.751), Caucasian (SMD = 0.58, 95% CI (− 0.06, 1.22), *P* = 0.077) and African population (SMD = 9.36, 95% CI (− 1.27, 19.99), *P* = 0.084). Stratification by sample size showed HCC group had significantly higher leptin levels than CFC group in both small (*n* < 100) sample numbers (SMD = 1.57, 95% CI (0.22, 2.91), *P* = 0.022) and large (*n* ≥ 100) sample numbers (SMD = 2.23, 95% CI (1.21, 3.26), *P* = 0.000). Stratification by mean age showed HCC group had significantly higher leptin levels than CFC group in both “< 60” (SMD = 2.87, 95% CI (1.57, 4.17), *P* = 0.000) and “≥ 60” (SMD = 0.76, 95% CI (0.03, 1.49), *P* = 0.040). Stratification by study design showed HCC group had significantly higher leptin levels than CFC group in both case-control studies (SMD = 3.81, 95% CI (1.83, 5.79), *P* = 0.000) and nested case-control studies (SMD = 0.14, 95% CI (0.01, 0.26), *P* = 0.035). Stratification by assay method revealed the HCC group had significantly higher leptin levels than the CFC group by both “ELISA” (SMD = 2.13, 95% CI (1.27, 2.99), *P* = 0.000) and “RIA” (SMD = 0.79, 95% CI (0.39, 1.19), *P* = 0.000). Stratification by Alanine aminotransferase (ALT) levels of HCC patients showed HCC group had significantly higher leptin levels than CFC group in “< 70 U/L” (SMD = 4.42, 95% CI (2.26, 6.50), *P* = 0.000), but not in the “≥ 70 U/L” (SMD = 0.43, 95% CI (− 0.38, 1.23), *P* = 0.296). Stratification by albumin levels of HCC patients showed HCC group had significantly higher leptin levels than CFC group in “< 3.5 g/dl” (SMD = 3.47, 95% CI (1.28, 5.66), *P* = 0.002), but not in the “≥ 3.5 g/dl” (SMD = 0.12, 95% CI (− 0.02, 0.26), *P* = 0.091).

**Fig. 4:**
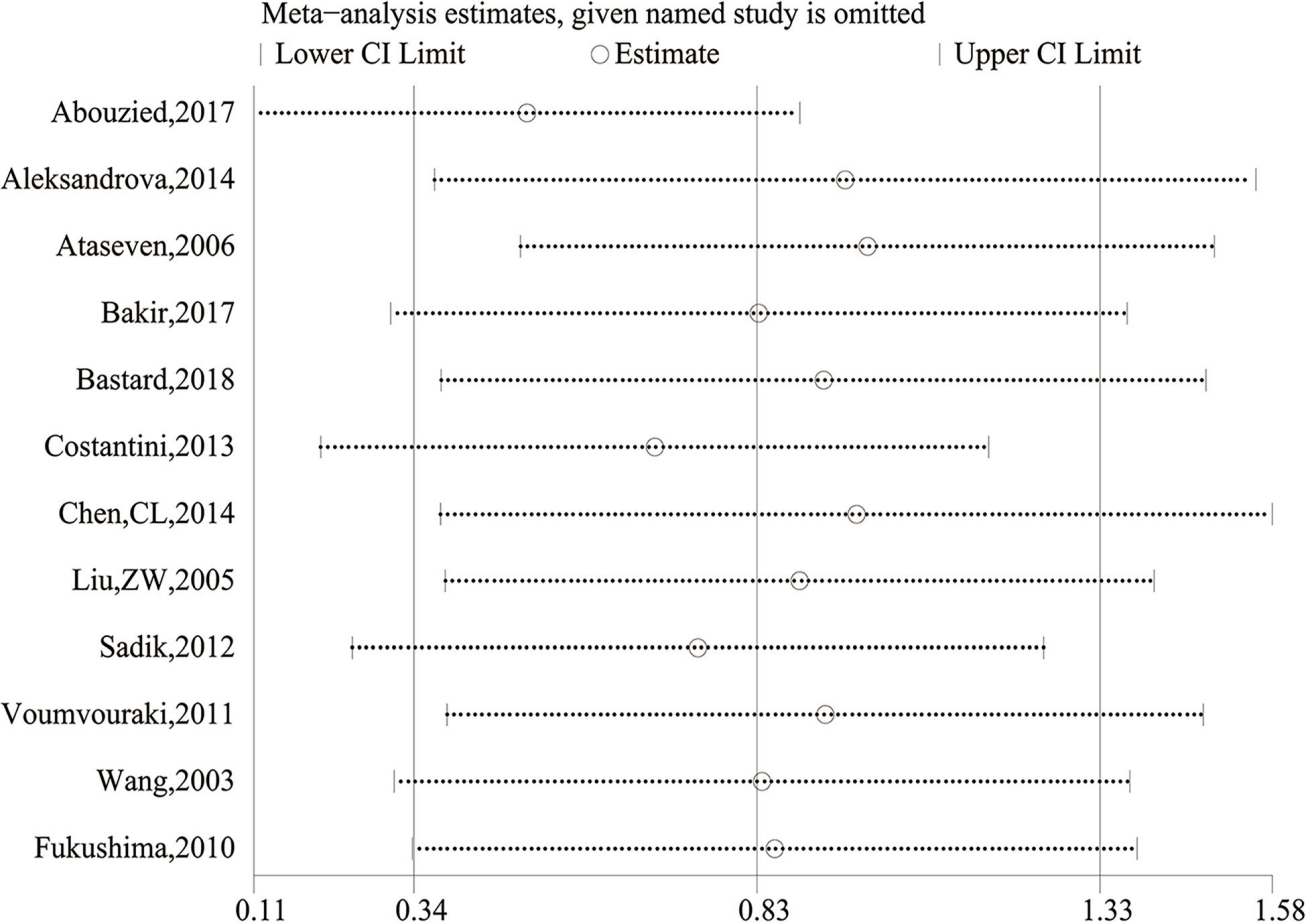
Sensitivity analysis of comparing circulating leptin levels between the HCC and cancer-free control group

Meta-regression analysis showed that only the ethnicity (*P* = 0.004), not the source of control (*P* = 0.242) and case (*P* = 0.185), sample size (*P* = 0.735), mean age (*P* = 0.420), study design (*P* = 0.344), assay method (*P* = 0.606), ALT (*P* = 0.172) and albumin (*P* = 0.853), had significant impacts on the heterogeneity in the meta-analysis. To assess the impacts of each study on the overall meta-analysis, we carried out sensitivity analysis using the leave-one-out method. No substantial change of data on leptin levels was observed. Therefore, the results of our meta-analysis were relatively stable and credible (Fig. 4).

**Fig. 5:**
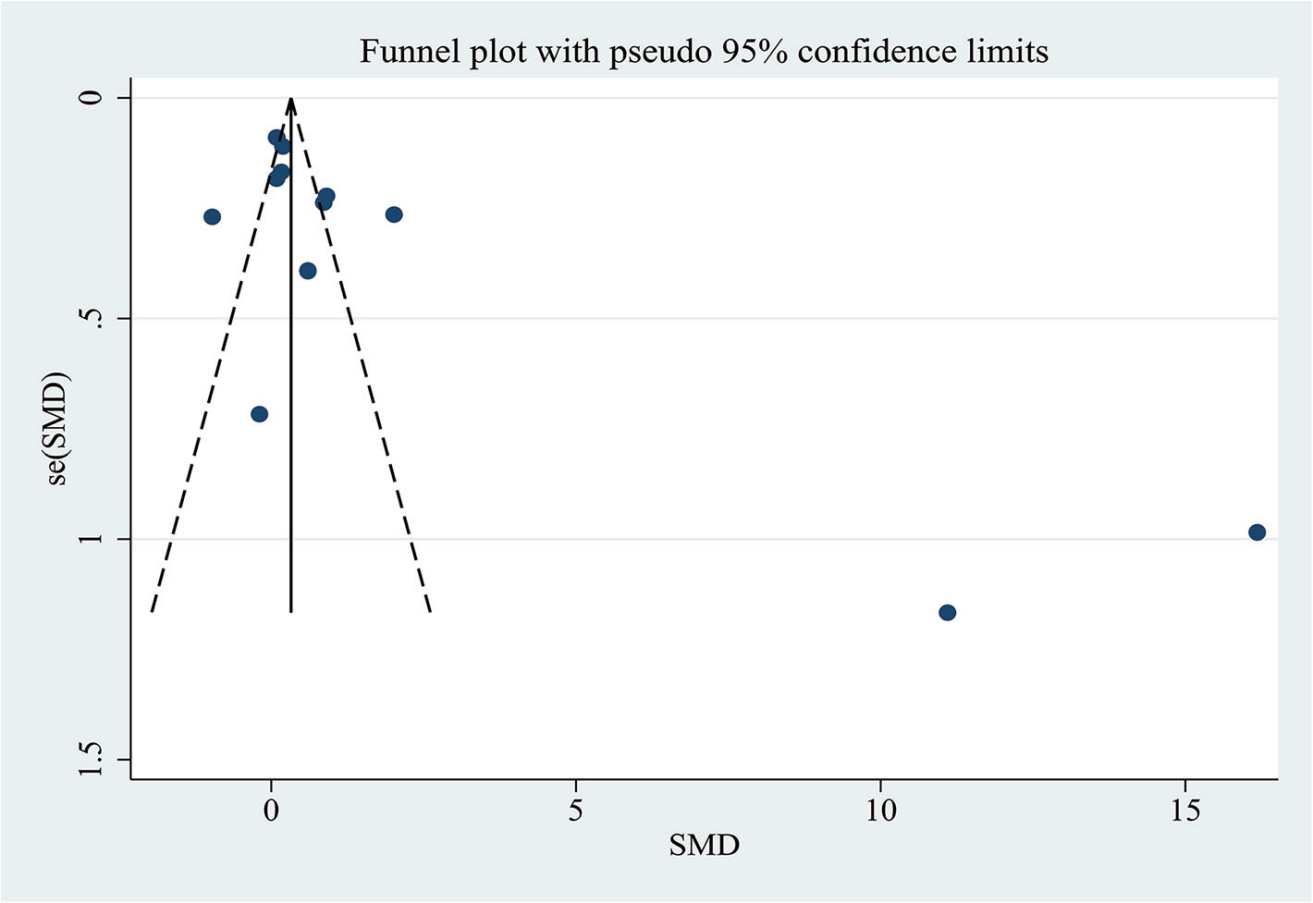
Funnel plot of comparing circulating leptin levels between the HCC and cancer-free control group

A funnel plot representing SMDs of the leptin levels in the HCC group compared to the CFC group was used to evaluate publication bias. Through the visual inspection of the funnel plot, there was obvious asymmetry that indicated a possibility of publication bias (Fig. 5), which were supported by Begg’s tests (*P* = 0.034) and Egger’s tests (*P* = 0.025). Therefore, further verification by trim and fill funnel plot was employed to adjust for the potential publication bias. However, the pooled data regarding leptin that had been significant before the adjustment with the “trim and fill” method remained significant after the adjustment (SMD = 3.486, 95% CI (0.937–6.035), *P* < 0.05), indicating that this publication bias did not affect the pooled estimates.

### Association between circulating AdipoQ levels and HCC risk

**Fig. 6:**
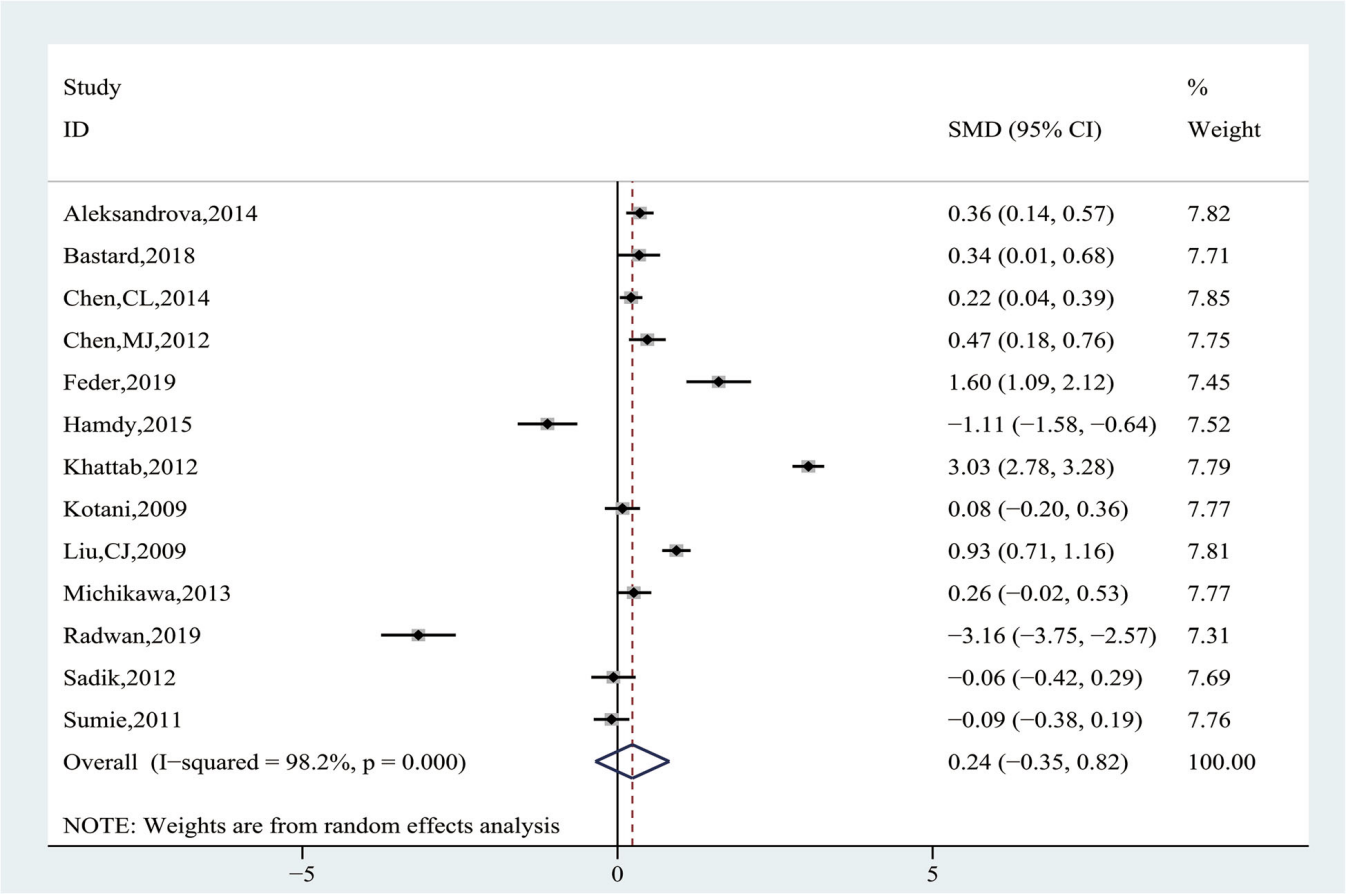
Forest plot of comparing circulating adiponectin levels between the HCC and cancer-free control group

Pooling data of 13 studies [21, 24–26, 28, 30–33, 35–37, 40] with 2092 participants were evaluated on the association between AdipoQ levels and HCC risk. Heterogeneity analysis showed significant heterogeneity among the studies (I^2^ = 98.2%, *P* = 0.000), and the random-effect model was applied. There was no statistical difference in HCC and CFC group on AdipoQ levels(SMD = 0.24, 95% CI (− 0.35, 0.82), *P* = 0.430) (Fig. 6).

**Fig. 7:**
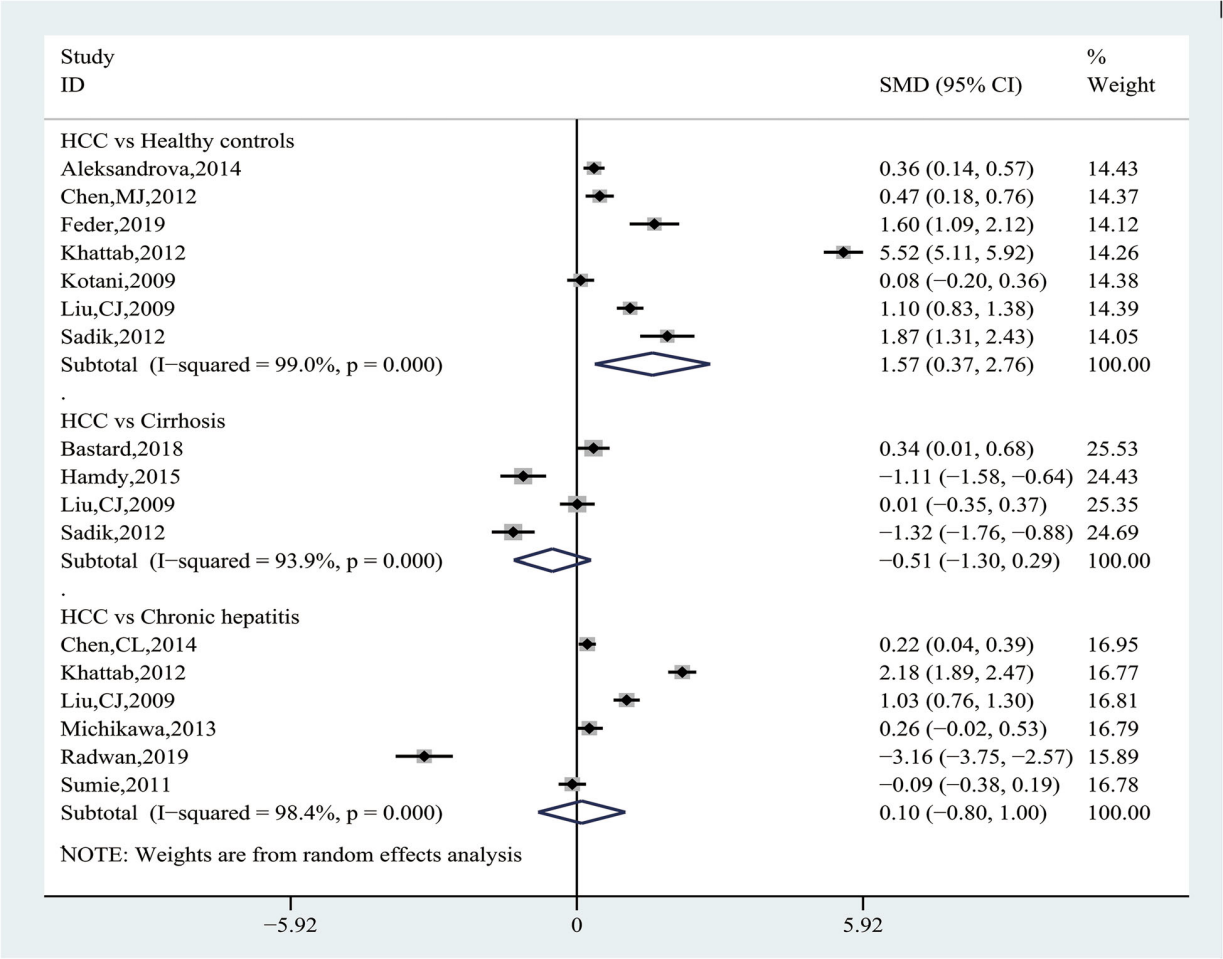
Forest plot of the subgroup analyses concerning circulating adiponectin levels

Subgroup analysis, according to the source of CFC group, showed HCC group had significantly higher AdipoQ levels than the healthy control group (SMD = 1.57, 95% CI (0.37, 2.76), *P* = 0.010), but there was no statistical difference compared with the chronic hepatitis group (SMD = 0.10, 95% CI (− 0.80, 1.00), *P* = 0.826) and the cirrhosis group (SMD = − 0.51, 95% CI (− 1.30, 0.29), *P* = 0.213) (Fig. 7 and Table 5). We further conducted subgroup analysis by the source of case group, and the results showed viral HCC had significantly higher AdipoQ levels than healthy controls (SMD = 1.11, 95% CI (0.44, 1.78), *P* = 0. 0.001), and HCV-related HCC had significantly lower AdipoQ levels than HCV-related cirrhosis (SMD = − 1.22, 95% CI (− 1.54, − 0.90), *P* = 0. 0.000), whereas there was no difference in other subgroups (Table 5).

**Table 5:** Subgroup analysis of the association between circulating adiponectin levels and HCC risk

Variable	Included studies	Test of association			Test of heterogeneity		
		SMD	95%CI	*P*-value*	Modal	*P*-value	I^2^
HCC vs Healthy controls							
Overall	[24, 29, 31, 34–36, 40]	1.57	0.37, 2.76	**0.010**	RE	0.000	99.0%
HCC(unreported reason) vs Healthy controls	[24, 31, 34, 35]	1.88	−0.31, 4.08	0.092	RE	0.000	99.5%
Viral HCC vs Healthy controls	[29, 36, 40]	1.11	0.44, 1.78	**0.001**	RE	0.000	90.8%
HCC vs Cirrhosis							
Overall	[27, 33, 36, 40]	−0.51	−1.30, 0.29	0.213	RE	0.000	93.9%
Viral cirrhotic HCC vs Viral cirrhosis	[27, 33]	−0.37	−1.80, 1.05	0.607	RE	0.000	95.9%
HCV-related HCC vs HCV-related cirrhosis	[33, 40]	−1.22	−1.54, −0.90	**0.000**	FE	0.531	0.0%
HCC vs Chronic hepatitis							
Overall	[28, 34, 36, 38, 39, 43]	0.10	−0.80, 1.00	0.826	RE	0.000	98.4%
HCC(unreported causes) vs Chronic hepatitis C	[34, 39]	− 0.48	−5.71, 4.75	0.857	RE	0.000	99.6%
HCV-related HCC vs Chronic hepatitis C	[28, 43]	0.08	−0.22,0.38	0.599	RE	0.068	70.0%
Viral HCC vs Chronic viral hepatitis	[28, 36, 38, 43]	0.35	−0.08, 0.78	0.108	RE	0.000	91.8%
Ethnicity							
Asian	[28, 29, 35, 36, 38, 43]	0.31	0.02, 0.61	**0.036**	RE	0.000	88.3%
Caucasian	[24, 27, 31]	0.73	0.11, 1.35	**0.022**	RE	0.000	90.3%
African	[33, 34, 39, 40]	−0.32	−2.93, 2.29	0.811	RE	0.000	99.5%
Sample size							
< 200	[27, 31, 33, 39, 40, 43]	0.76	0.03, 1.50	**0.042**	RE	0.000	98.5%
≥ 200	[24, 28, 29, 34–36, 38]	−0.40	−1.34, 0.54	0.403	RE	0.000	97.1%
Mean age							
< 60	[27–29, 33, 34, 36, 39, 40]	0.10	−0.85, 1.05	0.833	RE	0.000	98.8%
≥ 60	[24, 35, 43]	0.13	−0.14, 0.39	0.362	RE	0.037	69.7%
Study design							
Nested Case-control	[24, 27, 28, 35, 38]	0.25	0.14, 0.36	**0.000**	EE	0.585	0.0%
Case-control	[29, 34, 40, 43]	0.84	−0.74, 2.12	0.298	RE	0.000	99.2%
Cross-sectional	[33, 36, 39]	−1.10	−3.46, 1.26	0.361	RE	0.000	99.0%
Sample source							
Serum	[24, 27, 29, 31, 33–36, 39, 40, 43]	0.23	−0.51, 0.97	0.540	RE	0.000	98.5%
Plasma	[28, 38]	0.23	0.08, 0.38	**0.003**	FE	0.795	0.0%
Assay method							
ELISA	[24, 27, 28, 31, 33, 35, 36, 38–40, 43]	−0.03	−0.45, 0.40	0.901	RE	0.000	95.7%
Non-ELISA	[29, 34]	1.75	−0.75, 4.26	0.170	RE	0.000	99.4%
Alanine aminotransferase							
< 70 U/L	[28, 33, 36, 40, 43]	0.00	−0.53, 0.53	0.992	RE	0.000	94.8%
≥ 70 U/L	[27, 34, 39]	0.08	−2.96, 3.12	0.958	RE	0.000	99.5%
Albumin							
< 3.5 g/dl	[33, 34, 40]	0.62	−1.98, 3.22	0.639	RE	0.000	98.5%
≥ 3.5 g/dl	[27, 28]	0.24	0.09,0.40	**0.002**	RE	0.000	99.4%

*RE* Random-effects model, *FE* Fixed-effects model, *HCC* Hepatocellular carcinoma, *HCV* hepatitis C virus

*Statistically significant results were shown in bold

**Fig. 8:**
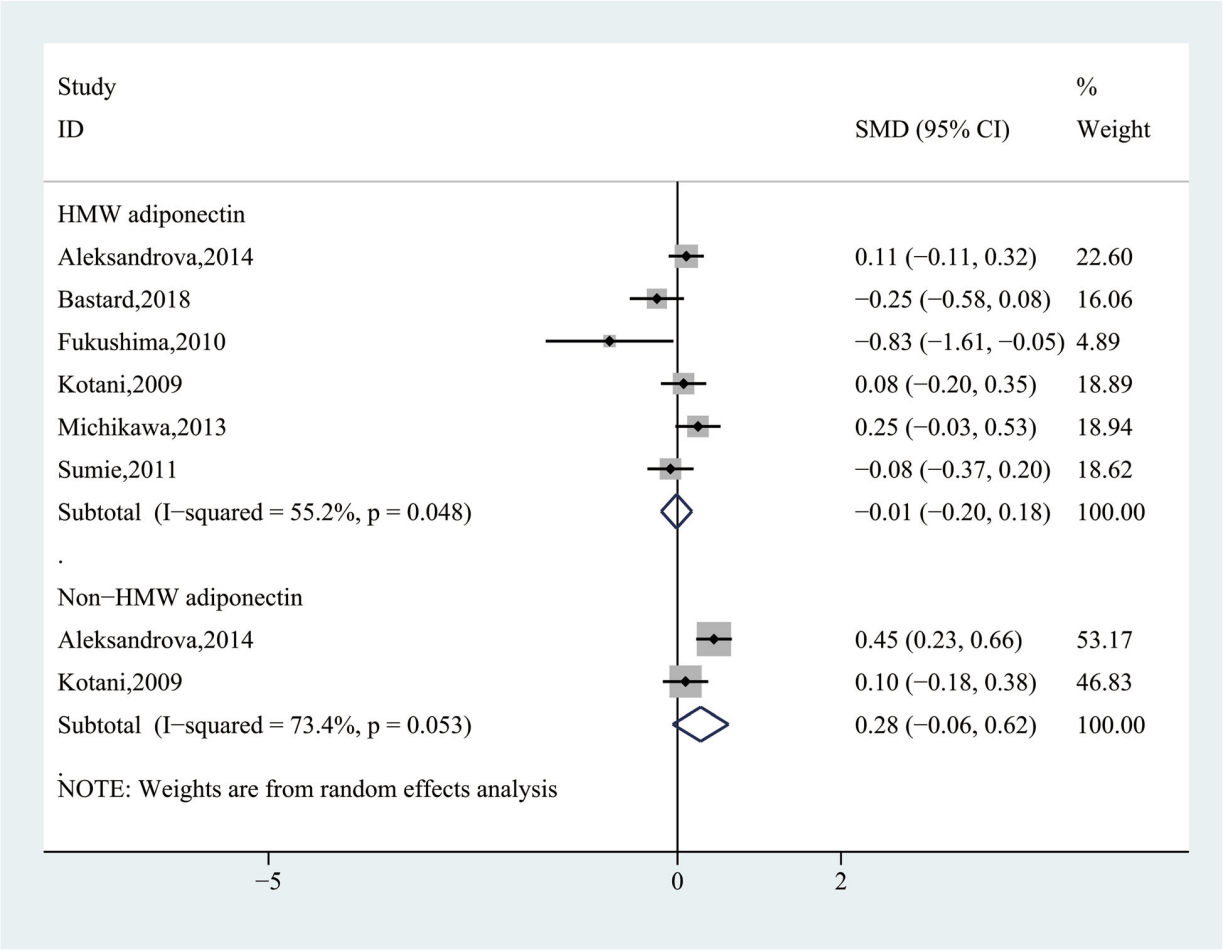
Forest plot of the subgroup analyses based on the molecular-weight of adiponectin between the HCC and cancer-free control group

Subgroup analysis, according to the molecular-weight of AdipoQ, showed no significant difference about high-molecular-weight AdipoQ (SMD = − 0.01, 95% CI (− 0.20, 0.18), *P* = 0.911) and non-high-molecular-weight AdipoQ (SMD = 0.28, 95% CI (− 0.06, 0.62), *P* = 0.103) levels in the HCC group and CFC group (Fig. 8). In addition, we also performed other subgroup analysis and the results were shown in Table 5. Stratification by ethnicity showed HCC group had significantly higher AdipoQ levels than CFC group in Asian (SMD = 0.31, 95% CI (0.02, 0.61), *P* = 0.036) and Caucasian population (SMD = 0.73, 95% CI (0.11, 1.35), *P* = 0.022), but not in African population (SMD = − 0.32, 95% CI (− 2.93, 2.29), *P* = 0.811). Stratification by sample size showed HCC group had significantly higher AdipoQ levels than CFC group in small (*n* < 200) sample numbers (SMD = 0.76, 95% CI (0.03, 1.50), *P* = 0.042), but not in large (*n* ≥ 200) sample numbers (SMD = − 0.40, 95% CI (− 1.34, 0.54), *P* = 0.403). Stratification by mean age showed no significant difference in the HCC group and CFC group in both “< 60” (SMD = 0.10, 95% CI (− 0.85, 1.05), *P* = 0.833) and “≥ 60” (SMD = 0.13, 95% CI (− 0.14, 0.39), *P* = 0.362). Stratification by study design showed HCC group had significantly higher AdipoQ levels than CFC group in nested case-control studies (SMD = 0.25, 95% CI (0.14, 0.36), *P* = 0.000), but not in case-control studies (SMD = 0.84, 95% CI (− 0.74, 2.12), *P* = 0.298), and cross-sectional studies (SMD = − 1.10, 95% CI (− 3.46, 1.26), *P* = 0.361). Stratification by the sample source revealed HCC group had significantly higher AdipoQ levels than CFC group in the source of plasma (SMD = 0.23, 95% CI (0.08, 0.38), *P* = 0.003), but not in the source of serum (SMD = 0.23, 95% CI (− 0.51, 0.97), *P* = 0.540). Stratification by assay method revealed no significant difference in the HCC group and CFC group by both “ELISA” (SMD = − 0.03, 95% CI (− 0.45, 0.40), *P* = 0.901) and “Non-ELISA” (SMD = 1.75, 95% CI (− 0.75, 4.26), *P* = 0.170). Stratification by ALT levels of HCC patients showed no significant difference in the HCC group and CFC group in both “< 70 U/L” (SMD = 0.00, 95% CI (− 0.53, 0.53), *P* = 0.992) and “≥ 70 U/L” (SMD = 0.08, 95% CI (− 2.96, 3.12), *P* = 0.958). Stratification by albumin levels of HCC patients showed HCC group had significantly higher AdipoQ levels than CFC group in “≥ 3.5 g/dl” (SMD = 0.24, 95% CI (0.09,0.40), *P* = 0.002), but not in the “< 3.5 g/dl” (SMD = 0.62, 95% CI (− 1.98, 3.22), *P* = 0.639). In addition, we also found that AdipoQ levels in HCC patients were not related to gender(man vs woman: SMD = − 0.29, 95% CI (− 0.69, 0.11), *P* = 0.153) and vascular invasion (present vs absent: SMD = 0.19, 95% CI (− 0.11, 0.49), *P* = 0.208).

Khattab et al. [30] found that AdipoQ levels in HCC with the size of nodules≥5 cm were significantly higher than<5 cm (24.2 ± 2.1 vs 20.8 ± 3.8, *P* = 0.009), whereas, AdipoQ levels were not related to TNM stages, number of nodules and lymph node metastasis. Feder et al. [27] discovered that AdipoQ levels were no statistical difference in HCC and colorectal liver metastases patients, and negatively related to steatosis grade, but not correlate with inflammation or fibrosis score. Sadik et al.^35^ reported that AdipoQ levels of cirrhotic HCC were significantly higher than the noncirrhotic HCC group, whereas leptin was not.

**Fig. 9:**
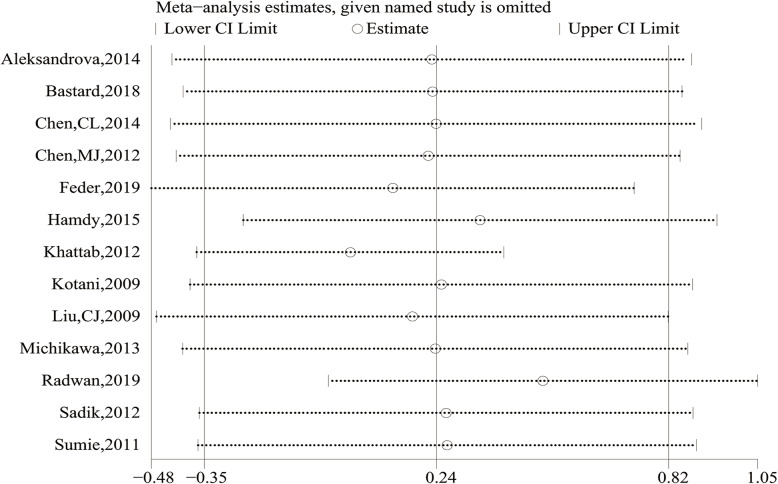
Sensitivity analysis of comparing circulating adiponectin levels between the HCC and cancer-free control group

Meta-regression analysis showed that the source of control (*P* = 0.150) and case (*P* = 0.579), ethnicity (*P* = 0.338), sample size (*P* = 0.140), mean age (*P* = 0.540), study design (*P* = 0.283), assay method (*P* = 0.092), source of sample (*P* = 0.993), ALT(*P* = 0.544) and albumin (*P* = 0.575) had no significant effects on the heterogeneity in the meta-analysis. We also carried out sensitivity analysis using the leave-one-out method, and no substantial change of data on AdipoQ levels were observed, therefore, the results of our meta-analysis were relatively stable and credible (Fig. 9).

**Fig. 10:**
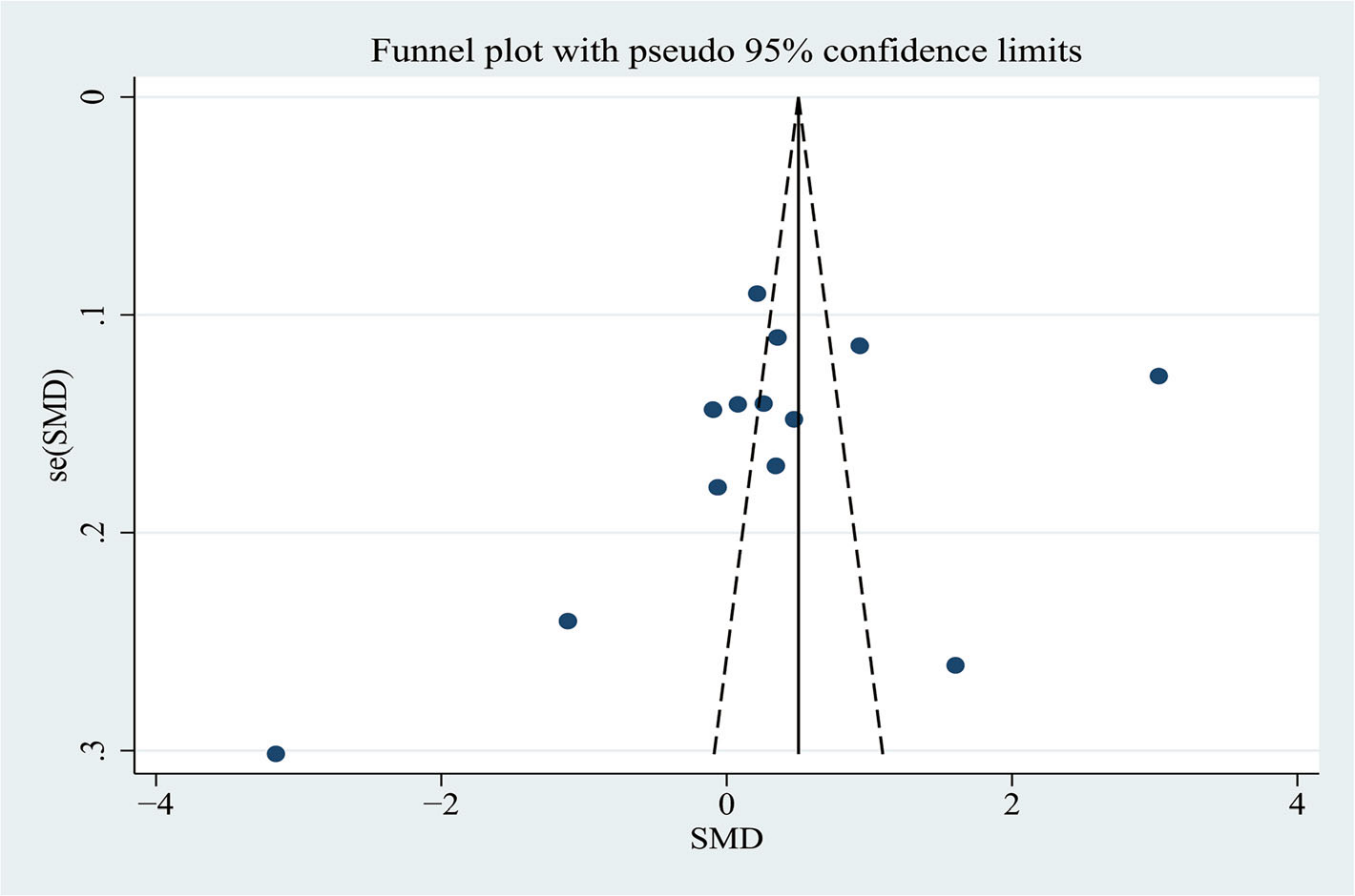
Funnel plot of comparing circulating adiponectin levels between the HCC and cancer-free control group

A funnel plot representing SMDs of the AdipoQ levels in the HCC group compared to the CFC group was used to assess publication bias. Through the visual inspection of the funnel plot, there was obvious asymmetry that indicated a possibility of publication bias (Fig. 10), which were not supported by the Begg’s tests (*P* = 0.300) and Egger’s tests (*P* = 0.142); therefore, further verification by trim and fill funnel plot was employed to adjust for the potential publication bias. The result of the “trim and fill” method revealed that no trimming was performed and the data was unchanged, suggesting that there was no significant publication bias.

### Association between leptin, AdipoQ gene polymorphism and HCC risk

**Fig. 11:**
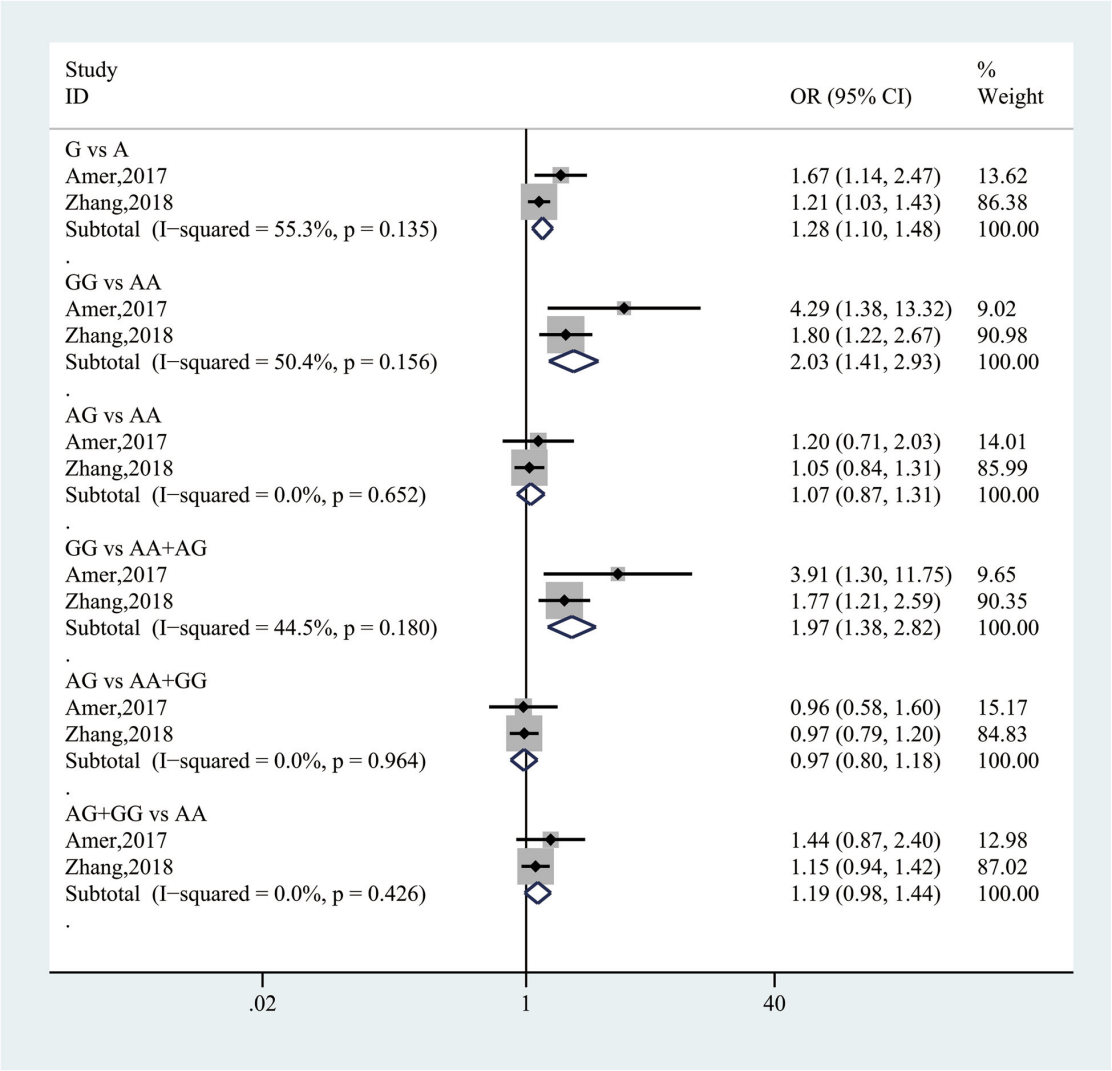
Forest plot for the association between leptin rs7799039 and HCC risk

Pooling data from 2 studies [41, 43] with 1746 participants evaluated the association between leptin rs7799039 and HCC risk. In the allele model analysis, the G allele was significantly associated with an increased risk in HCC (G vs A: OR = 1.28, 95% CI (1.10, 1.48), *P* = 0.002). In the codominant model analysis, the GG genotype was associated with a 2.03-fold elevated risk in HCC (GG vs AA: OR = 2.03, 95% CI (1.41, 2.93), *P* = 0.000), whereas the AG genotype was not (AG vs AA: OR = 1.07, 95% CI (0.87, 1.31), *P* = 0.505). In the recessive model analysis, the GG genotype was associated with a 1.97-fold increased risk in HCC (GG vs AA+AG: OR = 1.97, 95% CI (1.38, 2.82), *P* = 0.000). However, in the overdominant and dominant model analysis, the AG and AG + GG genotypes were no significantly associated with the risk in HCC (AG vs AA+GG: OR = 0.97, 95% CI (0.80, 1.18), *P* = 0.770; AG + GG vs AA: OR = 1.19, 95% CI (0.98, 1.44), *P* = 0.078). There was no significant heterogeneity in the above results (Fig. 11).

As for AdipoQ, Cai et al. [42] found that the AdipoQ rs1501299 was associated with the increased susceptibility to HCC, and the additive model showed that the GT and GG genotypes were significantly associated with an increased risk in HCC (GT vs TT: OR = 2.83, 95% CI (1.36, 5.89), *P* = 0.006; GG vs TT: OR = 4.52, 95% CI (2.25, 9.11), *P* = 0.001). In the dominant model analysis, the GG + GT genotypes were associated with a 3.8-fold elevated risk in HCC(GG + GT vs TT: OR = 3.795, 95% CI (1.92, 7.49), *P* = 0.001). However, the rs266729, rs822395, rs822396, and rs2241766 were not significantly associated with HCC. Unfortunately, we just retrieved one study that evaluated the association of AdipoQ gene polymorphism with HCC, so we failed to perform a related meta-analysis.

### Dose-response of circulating AdipoQ, leptin levels and HCC risk

**Fig. 12:**
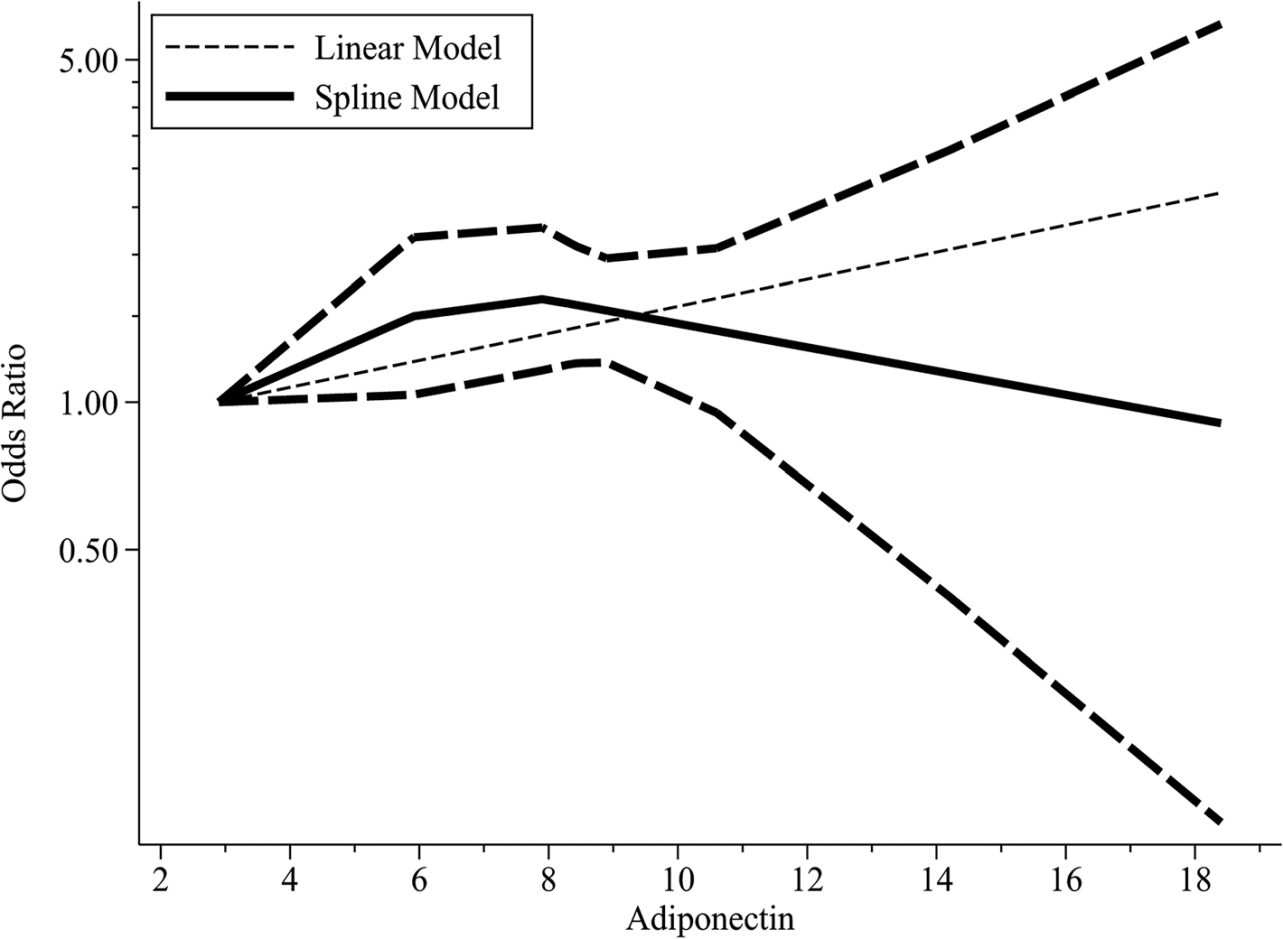
Dose-response associations of circulating adiponectin levels and HCC risk

Pooling data from 4 studies [21, 25, 32, 35] with 1507 participants showed that there was a linear dose-response relationship between circulating AdipoQ levels and HCC risk (_Pnon-linearity_ = 0.233). We defined the increment in 1 μg/ml AdipoQ levels as a unit to show the trend more clearly. The trends were significant for increasing HCC risk per one unit increase of AdipoQ (OR = 1.066, 95% CI (1.03, 1.11), *P* = 0.001; Fig. 12), without significant heterogeneity (P_heterogeneity_ = 0.338).

As for leptin, Aleksandrova et al. [21] and Chen et al. [25] both confirmed that circulating leptin levels were no significant dose-response trend in the development of HCC. Unfortunately, we only found two studies, and unable to perform a meta-analysis.

### Association between AdipoQ, leptin and survival in HCC

**Fig. 13:**
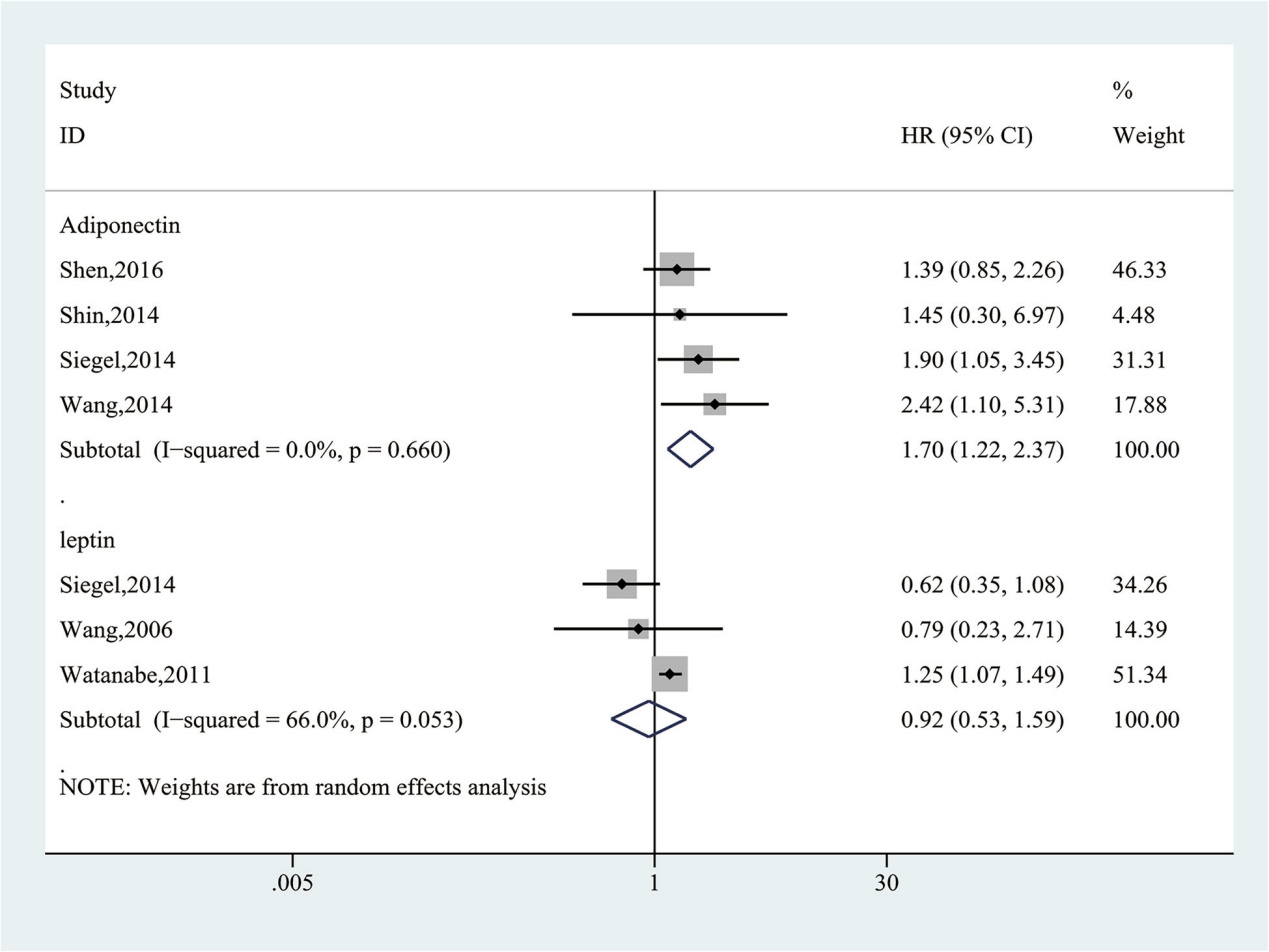
Forest plot of the relationship between adiponectin, leptin expression and survival in HCC

Pooling data of 4 studies [44–46, 48] with 435 participants analyzed the association between AdipoQ expression and survival in HCC. Heterogeneity analysis showed that no heterogeneity was observed among the studies (I^2^ = 0%, *P* = 0.660), the fixed-effect model was applied. The results showed that high/positive expression of AdipoQ was significantly related to lower overall survival (OS) in HCC patients (HR = 1.70, 95% CI (1.22, 2.37), *P* = 0.002; Fig. 13).

Pooling data of 3 studies [44, 47, 49] with 241 participants measured the association between leptin expression and prognosis for HCC. Heterogeneity analysis showed that significant heterogeneity was observed among the studies (I^2^ = 66.0%, *P* = 0.053), the random-effect model was applied. The results showed that high/positive expression of leptin was not significantly associated with prognosis in HCC patients (HR = 0.92, 95% CI (0.53, 1.59), *P* = 0.766; Fig. 13).

## Discussion

In the past two decades, many studies have explored the relationship between the abnormal expression of leptin and AdipoQ and various obesity-related cancers. In 2016, a meta-analysis of 107 articles [50] was conducted to investigate AdipoQ levels in various malignancies and found that AdipoQ levels in certain cancers (including acute leukemia, multiple myeloma, breast cancer, colorectal cancer, and uterine cancer, endometrial cancer, prostate cancer, thyroid cancer, tongue cancer, and gastroesophageal cancer) were significantly reduced, while HCC was significantly higher than the CFC group. However, the meta-analysis involved only 7 articles on HCC. Song et al. [51] analyzed 9 Chinese and English studies and got the same result. Our results showed that the AdipoQ levels of HCC patients were significantly higher than that of the healthy control group, but there is no significant difference in the AdipoQ levels compared with the CFC group. So far, no meta-analysis on leptin and liver cancer risk has been performed. Our results indicated that the leptin levels of liver cancer patients were significantly higher than that of the CFC group, healthy control group, and liver cirrhosis group. In addition, comparing the different sources of the HCC group and the CFC group, the results were different. Therefore, we can conclude that AdipoQ and leptin levels in patients with chronic hepatitis and cirrhosis have changed compared with healthy controls, which is consistent with Buechler’s conclusion [13].

Because the patients included in this study were recruited from different backgrounds of gender, race, other demographic parameters, and the overall health of the individual, a high degree of heterogeneity was observed in this meta-analysis, and the study results should be interpreted with caution. We performed meta-regression, subgroup analysis, and sensitivity analysis to determine the source of heterogeneity. The meta-regression results of leptin indicated that the heterogeneity came from race, and the subgroup analysis showed that the heterogeneity was related to the source of the control group, study design, assay method, and baseline albumin levels. In the pooled analysis of AdipoQ, the source of heterogeneity was not found by the meta-regression, and the heterogeneity was linked to the control group, study design, average age, and sample source by the subgroup analyses.

Many single nucleotide polymorphisms have been found in the leptin gene. The earliest is the LEP rs7799039 polymorphism, a SNP identified in the 50 untranslated regions of the leptin gene [52]. Research has been conducted on the tumor and recommendations have been made. It may affect the transcription levels and leptin expression [53]. Some previous meta-analysis showed that LEP rs7799039 polymorphism confers cancer risk [54–56]. However, no meta-analysis was performed to explore the association between LEP rs7799039 gene polymorphism and HCC risk. In this study, we found that the LEP rs7799039 polymorphism was related to the susceptibility to HCC. Unfortunately, too few studies were included, and conclusions should be confirmed by higher-quality studies.

Through dose-response, we can more clearly explore the relationship between AdipoQ and leptin and liver cancer risk. In 2019, Yoon et al. [57] found that AdipoQ levels were significantly associated with a decreased risk of breast cancer, colorectal cancer, and endometrial cancer, while leptin was associated with an increased risk of cancers such as endometrial cancer and kidney cancer. However, our results indicated that the increase in AdipoQ levels was of great significance for increasing the risk of HCC. There were only 2 dose-response studies between leptin and HCC risk, so the meta-analysis was abandoned.

Leptin and AdipoQ may not only be closely related to the occurrence of cancer, but closely associated with the prognosis of cancer. Our findings indicated that the high/positive expression of AdipoQ in liver cancer patients was significantly associated with decreased OS, which is similar to a meta-analysis of 10 studies, which revealed that increased AdipoQ expression is associated with poor prognosis in cancer patients (including HCC patients) [58]. It is worth noting that in this meta-analysis, the high/positive expression of leptin was not significantly associated with the prognosis of HCC patients.

High levels of leptin may play an important role in promoting cancer cell migration, proliferation, survival, and angiogenesis [59, 60]. This is achieved by activating the Janus kinase/signal transducer and activator of transcription, phosphatidylinositol 3-kinase, mitogen-activated protein kinase, and extracellular signal-regulated kinase signaling pathway [61, 62], which is considered related to oncogenes [63, 64].. Leptin can also promote the development of liver fibrosis, steatosis, and pro-inflammatory [4, 65]. In addition, Mittenbuhler et al. the leptin signal is found in obesity as the promoter of HCC [66].

Many studies have found that AdipoQ has significant anti-proliferative, anti-cancer activity, and anti-inflammatory effects [67]. Nazmy et al. have found that the tumor suppressor activity of AdipoQ can provide the body with more anti-HCC effects by preventing the decrease of p53 expression, the reactivation of TNF-related apoptosis-inducing ligand signals, and the induction of apoptosis pathways [68]. Al-Gayyar et al. revealed that AdipoQ can completely prevent the increase of HCC-induced sulfatase-2, improve HCC-induced tumor invasion markers, matrix metalloproteinase 9, syndecan-1 and fibroblast growth Factor-2 induced by HCC, decrease the expression of NF-κB and tumor necrosis factor α (TNF-α) induced by HCC, to achieve liver protection [69]. Manieri’s research shows that AdipoQ can activate two proteins in hepatocytes, p38α and AMP-activated protein kinase, which can prevent cell proliferation and damage tumor growth [70]. Our finding that elevated AdipoQ is associated with a high risk of liver cancer and poor prognosis seems to be contradictory to the above view. Some mechanisms have been proposed to resolve this contradiction. 1) AdipoQ resistance: Even if AdipoQ is expressed in large amounts, it may not be able to prevent poor prognosis caused by the down-regulation of AdipoQ receptors or dysfunction of AdipoQ signaling pathway. Many HCC patients suffer from cirrhosis or fibrosis, both of which are related to the down-regulation of AdipoQ receptors in the liver and the decrease of AdipoQ clearance rate, which leads to AdipoQ resistance state [71]. Similarly, the expression of AdipoQ could have been enhanced to compensate for the progression of HCC, but due to the overall deterioration of the patient’s physical condition, higher AdipoQ levels were ineffective. 2) AdipoQ stimulates AKT-mediated activation of cancer cells, which is an important predictor of low survival rate [48, 72].

Some limitations should be considered in this meta-analysis. First, due to the high degree of heterogeneity, some results should be interpreted with caution. Secondly, few studies have been conducted to explore the correlation between leptin, AdipoQ gene polymorphism and HCC risk. The results of LEP rs7799039 gene polymorphism and HCC risk need more confirmation. Third, we partially extracted HR from the survival curve of the original article, which may introduce some small errors. Finally, almost all studies in the meta-analysis measured leptin and AdipoQ levels only once, and did not show long-term changes in the course of HCC.

## Conclusions

This study showed that high leptin levels were associated with a higher risk of HCC, which may be a useful biomarker for early detection of HCC. The AdipoQ levels were directly proportional to the risk of HCC with a linear dose-response relationship and were associated with poor prognosis, which may be a potential biomarker for evaluating the prognosis of HCC.

## Data Availability

The data that support the findings of this study are available from the corresponding author upon reasonable request.

## Abbreviations

HCC: Hepatocellular carcinoma
HCV: Hepatitis C virus
HBV: Hepatitis B virus
CFC: Cancer-free control
AdipoQ: Adiponectin
SMD: Standardized mean difference
OR: Odds ratio
HR: Hazard ratio
CI: Confidence interval
NOS: Newcastle-Ottawa Scale
ALT: Alanine aminotransferase
ELISA: Enzyme-linked immunosorbent assay
RIA: Radioimmunoassay
OS: Overall survival
